# Assessing Decentering Capacity in Athletes: A Moderated Mediation Model

**DOI:** 10.3390/ijerph20043324

**Published:** 2023-02-14

**Authors:** Pierluigi Diotaiuti, Giuseppe Valente, Stefano Corrado, Stefania Mancone

**Affiliations:** Department of Human Sciences, Society and Health, University of Cassino and Southern Lazio, 03043 Cassino, Italy

**Keywords:** decentering, athletes, emotional regulation, coping styles, metacognition, mental training

## Abstract

Decentering has received more attention in sports literature as a self-regulating skill capable of significantly limiting episodes of mental block in competitive situations. This contribution depicts a comparative study conducted with 375 Italian national and international athletes. The objective was to evaluate athletes’ decentralization skills across different sports and levels of competition and test a mediation model of decentering in sports with coping and emotional balance variables. Pearson bivariate correlations, linear hierarchical regression, and simple mediation analysis were conducted for all main measures (The Decentering Sport Scale, The Emotion Regulation Questionnaire, and The Coping Orientations to Problems Experienced). Outputs reported significant associations with emotional regulation and coping styles. Mediation analysis confirmed the central mediating role of decentering capacity which has indirect effects on both the coping ability of problem solving (z-value = 2.986; *p* = 0.003) and cognitive reappraisal (z-value = 2.779; *p* = 0.005). Decentering acts as a mediator between an athlete’s positive attitude, problem-orientation ability, and management of emotions in competition through cognitive reappraisal. The study highlights the significance of evaluating and enhancing decentralization skills in order to establish specific action mechanisms, which are crucial for both peak performance and the athlete’s health.

## 1. Introduction

The traditional focus of interventions for improving athlete performance and health has been on the physical aspects. With new research suggesting that athlete well-being has a significant impact on injury risk and athletic performance, there has been an increased focus in recent years on the mental aspects of athlete health. Meditation practices based on mindfulness have been shown to improve both mental and physical health in a number of clinical populations. Additionally, studies have investigated how mindfulness can improve athletic performance, reduce injury risk, and possibly even increase the speed of injury recovery [1]. Researchers discovered that practicing mindfulness can enhance one’s capacity to control emotions, address emotional dysfunction, enhance thought patterns, lessen negative thinking, as well as enhance physical function and interpersonal relationships [2]. The acceptance of one’s situation is a general clinical benefit of mindfulness, and this can reduce the internal conflict that can occur when the expectations of how life should be are different from how life actually is.

One of the main aspects of mindfulness is the concept of “decentering”, which is defined as the ability to view thoughts, feelings, and physical sensations as transient mental events rather than as self-referential truths or facts. It is the capacity of the individual to not get caught up in (often negative) thoughts and to let them go. In sports psychology, this approach can aid athletes in controlling perceived pressure, performance anxiety, and even preventing a performance downturn [3].

Decentering, or the ability to step outside of one’s immediate experience and observing one’s self in the process of constructing the experience, has been described as a central process of change in cognitive therapies that aim to help clients become experientially aware of the role that their minds plays in the construction of their reality [4].

The concepts of mindfulness, decentering, cognitive defusion, and self-compassion are often used interchangeably, but in reality, they can be distinguished effectively; this is the result of several studies that have investigated these construct differences by separating the factors [5,6].

Athletes who can step back from their emotions and thoughts and view stressful situations as opportunities rather than threats are more likely to perform well under pressure [7]. For example, when faced with pre-race anxiety, decentering athletes may benefit from rephrasing their anxiety as “I think I’m feeling nervous right now” rather than “I’m nervous”, as this may reduce the negative effects of the latter statement.

In sport psychology, athletes are taught mental strategies to help them deal with a number of difficult challenges related to how their minds work [8,9]. Decreases in rumination also have been linked to improvements in well-being and decreased psychological distress, suggesting that mindfulness at the dispositional level may be a crucial component [10,11]. People may be able to relate to events more objectively, which could help them avoid becoming overly mentally and emotionally involved [12], and thus help them recognize unpleasant emotions that need to be controlled more quickly [13]. Other research has shown that positive cognitive reappraisal increases the feelings of positive emotions in archery athletes and enhances performance in experimental tasks [14,15], while expression suppression does not increase motor performance in table tennis [16]. Arousal reappraisal also helps golfers avoid “choking”, and to some extent, it ensures performance in putting tasks, similarly to cognitive reappraisal [17].

Some studies have conceptualised decentering and related constructs as traits [6,18,19,20], while other scholars have defined them as states [21,22,23]. However, it is also hypothesised that if activated repeatedly over time and in various contexts, the expression of this metacognitive process may become more stable or trait-like [24]. Evidence points to a continuum in the distribution of one’s ability to decenter from commonplace negative inner events [6,19,25]. People at the lower end of this spectrum are more inclined to concentrate on the themes of unpleasant inner events and to rely on this knowledge to influence their behavior and decision-making.

The Experiences Questionnaire was the first psychometric tool that was used to measure decentering [26]; it originally consisted of fourteen items to measure the decentering factor and an additional six rumination items designed to control for response bias. In previous studies on the development of decentering scales, initial findings indicated that decentering is positively related to mindfulness, cognitive reappraisal, positive affect, and life satisfaction, and negatively related to experiential avoidance, rumination, negative affect, depression, anxiety, stress, expressive suppression, brooding, and cognitive fusion [26,27].

In various studies with mediation analyses, it has been found that decentering can mediate the effect of mindfulness and cognitive reappraisal on anxiety symptoms [28,29], the effect of mindfulness on depressive symptoms [29,30], the effect of self-focus on negative thoughts in depression [31], and the effect of rumination on depression [11,12]. Furthermore, studies have shown that the mindfulness ring can be a mediator between mindfulness interventions and the Positive Mindset [32] and between meditation and personal values [33].

In consideration of these studies, we decided to conduct a study focusing on the decentering capacity of professional athletes in different disciplines. The research was guided by four main hypotheses:

(1) decentering ability may exhibit significant differences, attributable to individual characteristics such as age, gender, and competitive experience;
(2) decentering ability may show significant differences in relation to the type of sports discipline practiced;
(3) higher competitive levels correspond to higher decentering ability in athletes;
(4) decentering may be a positive mediating factor between coping skills and emotion regulation in the athletes.

## 2. Materials and Methods

### 2.1. Participants

In order to test the hypotheses of this study, a statistical power analysis was performed for sample size estimation. The effect size (ES) was set to 0.30, considered to be medium, using Cohen’s criteria. Using the G*Power 3.1 software (Heinrich Heine Universität Düsseldorf, Düsseldorf, Germany), with consideration of the effect size and what is considered appropriate for performing an intergroup comparison while taking into account the athletes’ gender and level of competition (regional, national, international), the minimum number of participants was preliminarily set at 281. In view of mediation testing, we took into account the following: the minimum number of participants for conducting linear multiple regressions including eight prediction variables, indicated to be 106 participants by G*Power; in the absence of a specific sampling calculator for mediation analysis, the optimal guideline number indicated in the literature, which is a sample size of 405 required for 0.8 power; the median sample size number of 341 derived from literature surveys about the methods used to test for mediation [34,35]. The sample was recruited in a non-probabilistic way through an open invitation. For the study, 375 professional athletes were involved, including 222 males (59.2%) and 153 (40.8%) females, with an average age of 26.3 (SD = 11.65). They were asked to join the study for free through the collaboration of various professional federations and teams. An email of involvement in the study was sent to the presidency of the various sports federations with a description of the research and a request to allow the technical group and coaches to forward it to their respective athletes. The athletes were invited to participate by clicking on a link provided for filling out a questionnaire which covered both the collection of general information of a biographical type and the athlete’s level of sports activity, and the self-report psychometric instruments that were chosen for the study. The inclusion criteria of the subjects in the study included being active competitive athletes at the time the study was conducted. No particular criteria regarding training years and athletic level were set. The research was carried out on three levels of competition: regional (n = 175; 46.7%), national (n = 139; 37.1%), and international (n = 61; 16.3%). The sports disciplines included in the study, as reported in Table 1, were football (n = 106; 28.3%) archery (n = 67; 17.9%), volleyball (n = 45; 12%), swimming (n = 45; 12%), martial arts (n = 32; 8.5%), fitness (n = 31; 8.3%), basketball (n = 25; 6.7%), and rowing (n = 24; 6.4%). Participants in the study voluntarily gave their consent to the confidential treatment of their data in aggregate form for scientific purposes only. In June and July 2022, a questionnaire with a general information section and a psychometric section was given out. By using a specific link, the tool allowed users to fill out forms online and collect data on the Questbase platform. The subjects were informed that the questionnaire would only be used for research and that it was anonymous. In accordance with the Declaration of Helsinki, each approached athlete provided written informed consent. The Institutional Review Board of the Department of Human Sciences, Society and Health, University of Cassino and Southern Lazio, has given its approval to the protocol.

### 2.2. Tools

*The Decentering Sport Scale* [36] consists of 12 items and a 5-point lickert scale (never true to always true) and assesses the one-dimensional construct of decentering in a sport context. The DSS was developed in accordance with the decentering construct used for the Experience Questionnaire [26], a questionnaire already validated and used in many contexts. Decentering in the validation work of the DSS scale was considered from both a behavioural and a cognitive perspective, i.e., individuals (a) cognitively differentiate their thoughts from the true self and truth and (b) cease the habitual reaction to their experiences through behaviour. DSS can be applied for the evaluation of decentering in sports contexts in order to explore the effectiveness of various mental training programmes and to test processes of change. The application of DSS for different types of interventions (e.g., CBT, relaxation, and mindfulness interventions) can also serve as a tool to elucidate the similarities and differences in the use of these programmes in athletes’ mental training [7]. The Italian translation of the original instrument was used, obtained through the back translation method and the support of two language experts. The verification of full comprehensibility of the items was carried out through confrontation with a group of 20 college students enrolled in the university sports center (CUS) of the local university. No translated item revealed problems related to its full comprehension. The measurement validity of the scale was assessed through a Confirmatory Factor Analysis (CFA), which confirmed the single-factor structure of the instrument and reported good fit indices: X^2^(49) = 87.076; CFI = 0.966; TLI = 0.954; RMSEA = 0.049; RMSEA 90% C.I. [0.032; 0.065]; *p*-value = 0.522. The scale also showed good overall internal consistency: Cronbach’s α = 0.83 [CIs 95% 0.795; 0.851]; McDonald’s ω = 0.83; [CIs 95% 0.805; 0.858].

*The Emotion Regulation Questionnaire* [37] is a 10-item self-report questionnaire comprised of two scales representing two distinct emotion regulation strategies: cognitive reappraisal (six items) and expressive suppression (four items). The instructions ask the subject questions about his or her emotional life, specifically how he or she controls (that is, regulates and manages) emotions. The ten items are rated from strongly disagree to strongly agree on a 7-point Likert scale. The Italian validation of the scale was used [38]. The scale showed good overall internal consistency in this study with respect to the two component subscales. Cognitive reappraisal: Cronbach’s α = 0.85 [CIs 95% 0.818; 0.870]; McDonald’s ω = 0.85; [CIs 95% 0.823; 0.874]; Expressive suppression: Cronbach’s α = 0.72 [CIs 95% 0.680; 0.761]; McDonald’s ω = 0.73; [CIs 95% 0.679; 0.774].

*The Coping Orientations to Problems Experienced* [39] is a 60-item questionnaire assessing 15 different coping strategies. The items are rated on a 4-point Likert scale ranging from “I usually don’t do it” to “I almost always do it”. In the Italian validation of the COPE-NVI tool [40], five main factors were defined: Social support, Avoidance strategies, Positive attitude, Problem solving, and Turning to Religion. The scale for this study showed good overall internal consistency with respect to the five subscales. Social support: Cronbach’s α = 0.90 [CIs 95% 0.884; 0.916]; McDonald’s ω = 0.91; [CIs 95% 0.891; 0.921]; Avoidance strategies: Cronbach’s α = 0.87 [CIs 95% 0.853; 0.893]; McDonald’s ω = 0.87; [CIs 95% 0.848; 0.890]; Positive attitude: Cronbach’s α = 0.77 [CIs 95% 0.727; 0.802]; McDonald’s ω = 0.87; [CIs 95% 0.726; 0.803]; Problem solving: Cronbach’s α = 0.83 [CIs 95% 0.804; 0.857]; McDonald’s ω = 0.84; [CIs 95% 0.813; 0.865]; and Turning to Religion: Cronbach’s α = 0.75 [CIs 95% 0.703; 0.749]; McDonald’s ω = 0.76; [CIs 95% 0.716; 0.799].

### 2.3. Data Analysis

Statistical analyses were performed using the package SPSS (IBM, Chicago, IL, USA) version 26 and JASP (JASP Team 2023 computer software) version 0.17, while the free software G*Power version 3.1.9.4 (RRID:SCR_013726) was used for sample calculation. The main analyses performed were: descriptive statistics about gender, age, and sport disciplines; Confirmatory Factor Analysis (CFA) to test the single-factor structure of DSS and relative main fit indices (CFI, TLI, and RMSEA); Pearson bivariate correlations for all main measures (The Decentering Sport Scale, The Emotion Regulation Questionnaire, and The Coping Orientations to Problems Experienced) significant at *p* < 0.005 and at *p* < 0.001, 2-tailed; Cronbach’s alpha and McDonald’s omega as scale reliability coefficients; independent-samples t-test to explore differences in decentering between males and females; Welch Anova one-way with post-hoc Games-Howell and *p* < 0.05 to explore significant differences within the eight sport disciplines. Linear hierarchical regression was used to identify the predictors of decentering. A simple mediation analysis was conducted to test the role of decentering capacity as a mediator between the coping and the emotional management skills of the athletes.

## 3. Results

Table 2 below reports overall DSS scores considering sport disciplines and athletes’ gender.

**Figure 1:**
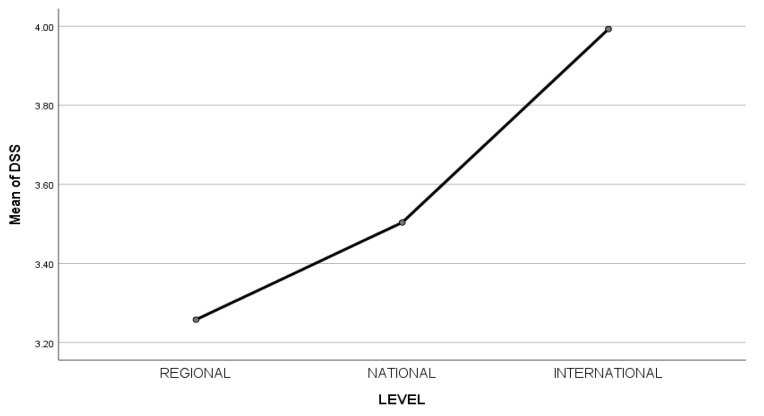
Mean of DSS in relation to the level of professionalism.

A one-way Welch ANOVA was conducted to determine if the ability to decenter (DSS score) was different for groups with different professionalism levels. Participants were classified into three groups: regional athletes (n = 175), national athletes (n = 139), and international athletes (n = 61). There were no outliers, and the data was normally distributed for each group, as assessed by boxplot and the Shapiro-Wilk test (*p* > 0.05), respectively. Homogeneity of variances was violated, as assessed by Levene’s Test of Homogeneity of Variance (*p* = 0.003). The DSS score was statistically significantly different between different professionalism groups; Welch’s (2, 166.508) = 42.058, *p* < 0.001. The DSS score increased from the regional (M = 3.26, SD = 0.54) to the national (M = 3.50, SD = 0.70) and international (M = 3.99, SD = 0.54) group, in that order. Games-Howell post hoc analysis revealed that the mean increase from regional group to national group (0.25, 95% CI [0.08, 0.42]) was statistically significant (*p* = 0.002), as well as the increase from national to the international group (0.49, 95% CI [0.27, 0.70], *p* = 0.000). As the competitive level increases (see Figure 1), the athlete’s capacity to decenter increases.

An independent-samples t-test was run to determine if there were differences in decentering capacity between males and females. There were no outliers in the data, as assessed by inspection of a boxplot. DSS scores for each level of gender were normally distributed, as assessed by Shapiro-Wilk’s test (*p* > 0.05), and there was homogeneity of variances, as assessed by Levene’s test for equality of variances (*p* = 0.289). Decentering was higher in male athletes (M = 3.55, SD = 0.63) than in female athletes (M = 3.35, SD = 0.67), with a statistically significant difference, M = 0.20, 95% CI [0.06, 0.33], t(373) = 2.883, *p* = 0.004. Taking only the international competition level into consideration, the difference between males (M = 4.09, SD = 0.48) and females (M = 3.85, SD = 0.59) loses statistic significance: M = 0.24, 95% CI [−0.03, 0.51], t(59) = 1.752, *p* = 0.085; Levene’s test: *p* = 0.517. In addition, no significant differences were found for decentering ability in relation to age, sport discipline, and years of competitive experience (*p* > 0.05).

Table 3 below reports bivariate correlations between the variables used in the study.

As seen in Table 3, the decentering measure strongly correlated (*p* ≤ 0.001) with the coping variables Positive Attitude and Problem Solving. Moreover, the decentering variable was positively correlated with the Cognitive Reappraisal variable with regard to emotion regulation. Decentering, on the other hand, was negatively correlated with the Turning to Religion variable.

As indicated above, a hypothesis of the study included assessing the mediating role of decentering within the athlete’s coping and emotional regulation strategies. Given that in the performed study the correlations recorded with the decentering variable showed a significant magnitude (>0.30) with Positive Attitude, Problem Orientation and Cognitive Reappraisal, we preliminarily identified these as component factors of the mediation model. Since it has been abundantly emphasized in the literature that a positive attitude can assist athletes in remaining motivated while avoiding anxiety and becoming overwhelmed [41,42], we set this as the main reference variable in the model and considered that decentering could significantly and positively mediate the effects of positive attitude on the ability to stay focused on the addressing the problem situation and on the functional control of the emotional sphere.

**Figure 2:**
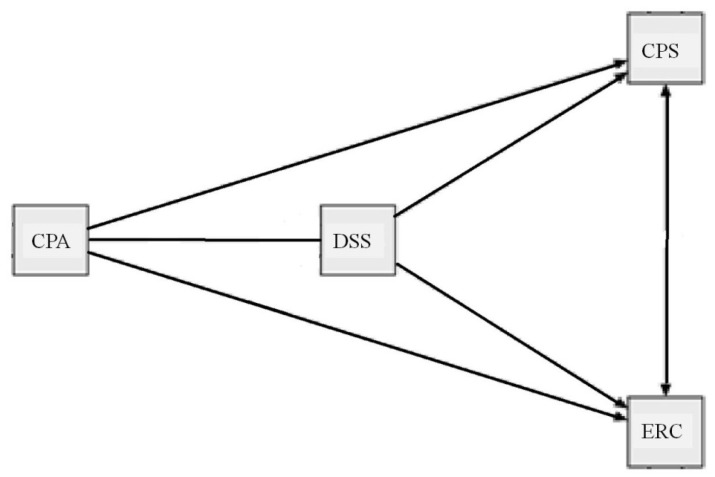
Mediation model hypothesis. Note: CPA—Positive attitude; DSS—Decentering Sport Scale; CPS—Problem solving; ERC—Cognitive Reappraisal.

A simple mediation model was tested in which decentering acted as a mediator between the athlete’s positive attitude, problem-orientation ability, and the management of emotions in competition through cognitive reappraisal (see Figure 2).

**Figure 3:**
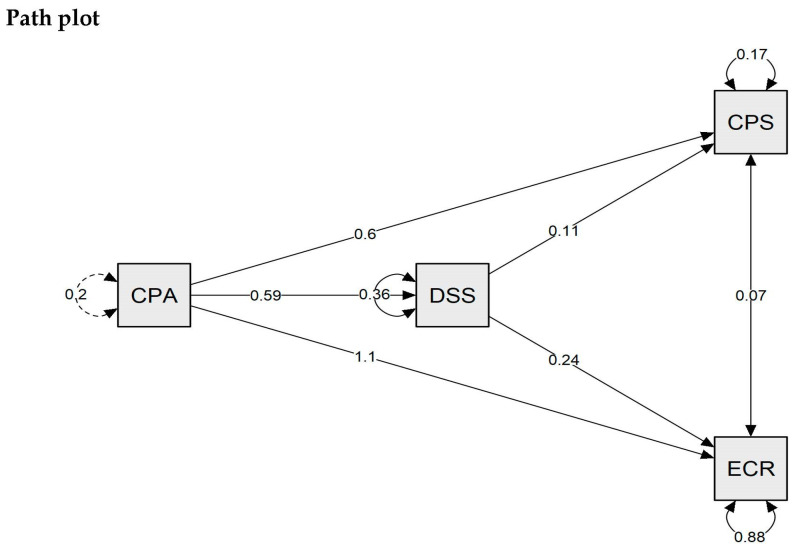
Mediation Analysis. Note: CPA—Positive attitude; DSS—Decentering Sport Scale; CPS—Problem solving; ERC—Cognitive Reappraisal.

The mediation analysis confirmed the central mediating role of decentering capacity, which acts with indirect effect on both the coping ability of problem solving (z-value = 2.986; *p* = 0.003) and the cognitive reappraisal (z-value = 2.779; *p* = 0.005). The direct effect and the total effect of the positive attitude variable were strongly significant (see Figure 3 below). The following Table 4, Table 5 and Table 6 show parameter estimates with details of direct effects, indirect effects, and total effects, respectively.

## 4. Discussion

The study highlighted the role of decentering capacity in sport. In all sport disciplines considered in this study, decentering capacity was shown to be significantly related to the level of competition. In particular, international level athletes showed a greater ability to decenter. Presumably, this capacity allows athletes to avoid being influenced by thoughts during competition and to be more oriented and focused on the action to be performed [43,44,45]. These results are in line with what has been observed in previous studies [46] which found that athletes’ flow state scores were higher when they were more mindful, had a clear goal, focused their attention, and felt in control. In our study, decentering has been shown to be an important factor in coping with the athlete’s difficulties in competition. Correlation analyses, showed important relationships between decentering skills and the coping subscales of Problem solving and Positive Attitude. Furthermore, decentering could play a role in emotional management in that during the performance, those who managed to decenter more were also those who managed their emotions better through cognitive reappraisal. There are many different strategies for controlling emotions, but cognitive reappraisal is a significant and useful one [37,47,48]. According to studies, suppression of expression is not positively correlated with motor performance, whereas positive cognitive reappraisal is [14]; this boosts athletes’ feelings of fulfillment and enhances their performance on experimental tasks [15]. In addition, the cognitive reappraisal of emotions is often associated with positive aspects of the athlete’s well-being [49,50]. A recent contribution on archery athletes highlighted that development of sport confidence and attentiveness is crucial to the results to be achieved [51]. A mediation analysis revealed a complexity in the relationship between cognitive reappraisal and archers’ performance, but it should be emphasised that athletes who directed attentional resources to cognitive reappraisal were found to have higher levels of sports confidence.

In the mediation model presented, it was found that an athlete who prepares for the competition with a positive attitude can manage his or her emotions better and can also be more oriented towards problem solving during the competition. Interestingly, decentering does not promote a reaction of suppression of emotions but rather helps athletes to activate a reinterpretation of them, improving their impact towards positive aspects.

The concept of positive attitude can be compared to that of self-confidence and self-efficacy in sport. Many studies and reviews have emphasised the importance of these factors for improving performance [52,53,54]. Athletes with more self-confidence are more likely to see anxiety as a functional sign and use it to improve their performance [55,56,57,58].

The action of positive attitude can be mediated by good decentering work on the part of the athlete. The ability to detach oneself from thoughts, whether positive or negative, combined with the athlete’s positive and winning attitude, can improve concentration and action orientation. These characteristics could be used together to define a model of intervention and/or coaching for professional athletes to help improve performance and concentration in competition. In the past few years, a number of studies have found that mindfulness and acceptance and commitment therapy can be used to improve the flow and performance of athletes. Clinical psychology has demonstrated the value of mindfulness training. College students’ levels of anxiety and depression can be reduced by mindfulness practice, which is widely used to control emotions and relieve stress [59,60,61]. Additionally, acceptance-based intervention techniques are used to raise athletes’ performance levels, demonstrating the value of improving athletes’ flow state experiences in the context of sports [57,62,63,64]. Furthermore, mindfulness training might enhance athletes’ sporting experiences while also providing psychological benefits [65,66].

This work should of course be seen in the light of some limitations. One limitation of the study concerns the fact that the selected athletes were not asked beforehand whether or not they had previously conducted courses or training on decentering or mindfulness skills. Furthermore, the sample was recruited in a non-propabilistic way by an open invitation to the athletes; the final number of participants was somewhat lower than the optimal number of 405 indicated for similar mediation analyses, but nevertheless, higher than the median sample size used in other studies reported in the literature. It should be also taken into account that the psychological measures used are obtained from participants’ self-report assessments. Future studies should therefore include the use of objective detection instruments for mental states associated with defocusing and emotional control under competitive pressure conditions.

## 5. Conclusions

In relation to the four hypotheses that guided the research, for the first, the results confirmed that the athletes’ gender is associated with different decentering ability which is significantly greater in males. However, this difference was not found to be attributable to age or years of competitive experience. As for the second hypothesis, no influence related to the type of discipline practiced was recorded. The results supported the third hypothesis, namely that as the competitive level increases, the ability to decentralize increases. For the international level, the gender difference shown in the overall sample loses significance. The model of positive mediation between coping skills and emotion regulation, which was the subject of the fourth hypothesis, also found clear confirmation in the analyses conducted. Overall, the present study shows the role of decentering as a characteristic to bring further attention on for the development of specific training with the support of experimental studies. These trainings may enable athletes to improve their ability to detach themselves from thoughts and be more concentrated during the match. Data indicate that more emphasis should be placed on decentralization capacity enhancement in female athletes, especially at the regional and national levels. Trainings to improve decentering ability in athletes could also be effective in assisting better management of pain states both during competition and in the period of recovery from injury [67,68,69,70]. In a larger direction, mindfulness can become a significant element in integrating and enhancing the athlete’s situational awareness, improving not only performance but helping to preserve his/her mental well-being through a positive, goal-oriented approach.

## Figures and Tables

**Table 1:** Descriptive Statistics.

Sport Disciplines	Frequency	Percent	C. Percent
Archery	67	17.9	17.9
male	41	61.2	
female	21	38.8	
Basketball	25	6.7	24.5
male	20	80.0	
female	5	20.5	
Martial Arts	32	8.5	33.1
male	22	68.8	
female	10	31.3	
Football	106	28.3	61.3
male	78	73.6	
female	28	26.4	
Fitness	31	8.3	69.6
male	19	61.3	
female	12	30.7	
Volleyball	45	12.0	81.6
male	10	22.2	
female	35	77.8	
Rowing	24	6.4	88.0
male	15	62.5	
female	9	37.5	
Swimming	45	12.0	100.0
male	17	37.8	
female	28	62.2	
Total	375	100.0	
male	222	59.2	
female	153	40.8	

**Table 2:** DSS scores considering sport discipline and athletes’ gender.

Sport Disciplines	Gender	Mean	S.D.	Min	Max	IC 95% Lower	Higher
Archery	male	3.69	0.57	2.44	4.78	3.50	3.86
	female	3.34	0.60	2.11	4.11	3.11	3.57
	total	3.55	0.60	2.11	4.78	3.40	3.70
Basket	male	3.45	0.51	2.67	4.44	3.22	3.68
	female	2.84	0.69	2.11	3.78	2.19	3.47
	total	3.33	0.58	2.11	4.44	3.09	3.56
Martial Arts	male	3.69	0.68	2.44	4.89	3.39	3.98
	female	3.53	0.75	2.11	4.56	3.05	3.98
	total	3.64	0.70	2.11	4.89	3.40	3.88
Football	male	3.42	0.68	1.00	5.00	3.28	3.50
	female	3.33	0.83	1.89	4.67	2.99	3.63
	total	3.40	0.72	1.00	5.00	3.26	3.53
Fitness	male	3.48	0.50	2.67	4.11	3.26	3.72
	female	3.42	0.70	2.11	4.11	3.00	3.80
	total	3.46	0.57	2.11	4.11	3.26	3.66
Volleyball	male	3.53	0.47	2.89	4.00	3.24	3.82
	female	3.44	0.65	1.56	4.56	3.21	3.65
	total	3.46	0.61	1.56	4.56	3.26	3.64
Rowing	male	3.72	0.49	3.11	4.44	3.47	3.97
	female	3.36	0.48	2.56	4.00	3.03	3.68
	total	3.59	0.51	2.56	4.44	3.38	3.81
Swimming	male	3.63	0.86	1.89	5.00	3.22	4.03
	female	3.26	0.62	2.22	4.67	3.05	3.49
	total	3.40	0.73	1.89	5.00	3.19	3.62
Total Sample	male	3.55	0.63	1.00	5.00	3.46	3.63
n = 375	female	3.35	0.67	1.56	4.67	3.25	3.45
	total	3.47	0.65	1.00	5.00	3.40	3.53

**Table 3:** Pearson’s Correlations.

Variable	1 DSS	2 CSS	3 CAS	4 CPA	5 CPR	6 CTP	7 EES	8 ECR
1. DSS	-							
2. CSS	0.014	-						
3. CAS	−0.089	0.187 ***	-					
4. CPA	0.401 ***	0.218 ***	−0.052	-				
5. CPR	0.355 ***	0.301 ***	−0.138 **	0.583 ***	-			
6. CTP	−0.221 ***	0.022	0.105 *	−0.265 ***	−0.141 **	-		
7. EES	0.065	−0.507 ***	0.122 *	0.042	−0.067	0.075	-	
8. ECR	0.316 ***	0.029	−0.160 **	0.489 ***	0.424 ***	−0.167 **	0.140 **	-

Note: DSS—Decentering Sport Scale; CSS—Social support; CAS—Avoidance strategies; CPA—Positive attitude; CPS—Problem solving; CTP -Turning to Religion; ECR—Cognitive Reappraisal; EES—Expressive Suppression. Correlation is significant: * *p* < 0.05, ** *p* < 0.01, *** *p* < 0.001.

**Table 4:** Direct effects.

							95% Confidence Interval	
			Estimate	Std. Error	z-Value	*p*	Lower	Upper
CPA	→	CPS	1.184	0.102	11.622	<0.001	0.984	1.383
CPA	→	ERC	0.972	0.110	8.874	<0.001	0.758	1.187

Note. Delta method standard errors, normal theory confidence intervals, ML estimator. CPA—Positive attitude; DSS—Decentering Sport Scale; CPS—Problem solving; ERC—Cognitive Reappraisal.

**Table 5:** Indirect effects.

									95% Confidence Interval	
					Estimate	Std. Error	z-Value	*p*	Lower	Upper
CPA	→	DSS	→	CPS	0.130	0.044	2.986	0.003	0.045	0.216
CPA	→	DSS	→	ERC	0.129	0.047	2.779	0.005	0.038	0.220

Note. Delta method standard errors, normal theory confidence intervals, ML estimator. CPA—Positive attitude; DSS—Decentering Sport Scale; CPS—Problem solving; ERC—Cognitive Reappraisal.

**Table 6:** Total effects.

							95% Confidence Interval	
			Estimate	Std. Error	z-Value	*p*	Lower	Upper
CPA	→	CPS	1.314	0.095	13.896	<0.001	1.129	1.499
CPA	→	ERC	1.102	0.102	10.850	<0.001	0.903	1.301

Note. Delta method standard errors, normal theory confidence intervals, ML estimator. CPA—Positive attitude; DSS—Decentering Sport Scale; CPS—Problem solving; ERC—Cognitive Reappraisal.

## Data Availability

The data presented in this study are available on request from the corresponding author.
